# The prevalence of diabetes and its association with comorbidities, demographic and behavioral factors: a cross-sectional survey among attendees of primary healthcare centers in Riyadh

**DOI:** 10.3389/fcdhc.2026.1796490

**Published:** 2026-05-25

**Authors:** Seema Nasser, Mamdouh M. Shubair, Mariam A. A. El-Metwally, Awad Alshahrani, Lubna Alnaim, Amani Alharthy, Mona Ibrahim Bin Amer, Faris Fatani, Raed Aldahash, Khadijah Angawi, Badr F. Al-Khateeb

**Affiliations:** 1 Department of Nursing, College of Nursing, King Saud bin Abdulaziz University for Health Sciences, Riyadh, Saudi Arabia; 2 King Abdullah International Medical Research Centre, Riyadh, Saudi Arabia; 3 Ministry of National Guard - Health Affairs, Riyadh, Saudi Arabia; 4 Department of Health Sciences program, University of Northern British Columbia (UNBC), Prince George, BC, Canada; 5 Department of Psychology and Global One Health Academy, North Carolina State University, Raleigh, NC, United States; 6 College of Medicine, King Saud bin Abdulaziz University for Health Sciences, Riyadh, Saudi Arabia; 7 College of Applied Medical Sciences, King Saud Bin Abdulaziz University for Health Sciences, Al Ahsa, Saudi Arabia; 8 Department of Health Science, College of Health and Rehabilitation Sciences, Princess Nourah bint Abdulrahman University, Riyadh, Saudi Arabia; 9 Department of Population Health Cluster, Riyadh Second Health Cluster, Riyadh, Saudi Arabia; 10 Riyadh Second Health Cluster, Riyadh, Saudi Arabia; 11 Department of Health Services and Hospital Administration, Faculty of Economics and Administration, King Abdulaziz University, Jeddah, Saudi Arabia

**Keywords:** comorbidities, prevalence, risk factors, Saudi Arabia, type 2 diabetes

## Abstract

**Background:**

Type 2 Diabetes (T2D) has been found to affect a quarter of the population in Saudi Arabia, a higher burden than worldwide. Given that there is a lack of robust epidemiological evidence regarding T2D among Saudi population, a need assessment is necessary to identify risk factors of diabetes. Hence, this study aimed to estimate prevalence of diabetes and its demographic and behavioral risk factors among Saudi Arabian population.

**Methods:**

A cross-sectional survey using a validated and reliable questionnaire was conducted from March to July 2023 In Riyadh, Saudi Arabia. Altogether, 14,239 patients, aged 18 years or older were selected from 48 primary healthcare centers. SPSS version 26.0 was used to analyze data.

**Results:**

About 12.4% of Saudi residents were diabetic and 11% were hypertensive. Compared to younger participants of <50 years, those ≥75 years older were 6.30 times more likely to be diabetic (AOR: 6.30; 95% CI: 5.11, 7.77). The risk of T2D was higher among males and obese individuals by 33% (AOR: 1.33; 95% CI: 1.17, 1.51) and 39% (AOR: 1.39; 95% CI: 1.11, 1.75), respectively. Compared to employed Saudi residents, unemployed residents were 1.49 times more likely to be diabetic (AOR: 1.49; 95% CI: 1.30, 1.70). Hypertensive patients were 10.42 times more likely to experience diabetes than non-hypertensive patients (AOR: 10.42; 95% CI: 9.02, 12.03). Patients with high cholesterol were 3.13 times more likely to develop diabetes than patients with low cholesterol levels (AOR: 3.13; 95% CI: 2.64, 3.72).

**Conclusion:**

The prevalence of diabetes appears to be high in Saudi Arabia. Older-aged males and unemployed Saudi residents were at higher risk of developing Diabetes than younger, employed, and female residents. Hypertension, obesity, and high cholesterol were other strong risk factors for T2D. Education was found to be a protective factor for Diabetes among Saudi population.

## 1 Introduction

Type 2 Diabetes (T2D), a growing global epidemic, is one of the main causes of mortality across the globe (1). The global prevalence of T2D has quadrupled over the last three decades, now affecting approximately one in eleven adults (2). In Saudi Arabia, the burden is particularly acute; the International Diabetes Federation (IDF) 2024 estimates a national prevalence of 23.1% among adults, one of the highest rates globally (3–5). This high prevalence escalates the risk of severe complications, including nephropathy, neuropathy, retinopathy, hypertension, stroke, and lower-limb amputations (6, 7), positioning T2D as a critical public health priority for the Kingdom.

The etiology of T2D is multifactorial. Established risk factors within the Saudi context include advanced age, dyslipidemia (high cholesterol and triglycerides), hypertension, and lifestyle behaviors such as tobacco use (8, 9). Recent meta-analytical evidence suggests that the prevalence of prediabetes is also rising significantly, creating a massive pool of individuals at high risk for progressing to clinical T2D (5). However, existing research often evaluates these factors within gender-stratified cohorts, resulting in a lack of comprehensive, population-level data that integrates sociodemographic, clinical, and behavioral variables (10, 11).

Given the scarcity of integrated epidemiological evidence, a robust assessment is required to identify the contemporary risk factors driving the diabetes epidemic in Saudi Arabia. Such evidence is essential for informing public health policies and strategically planning population-level interventions, particularly within the framework of Saudi Vision 2030. This study aims to determine the prevalence of diabetes and identify its associated risk factors among attendees of primary healthcare centers in Riyadh. We hypothesize that identifying these common determinants will provide the empirical foundation necessary to design targeted interventions and mitigate the adverse clinical outcomes associated with the disease.

## 2 Methods

### 2.1 Study design and target population

A cross-sectional survey using a validated and reliable questionnaire was conducted from March to July 2023 in Riyadh, Saudi Arabia. Patients who visited primary healthcare facilities in Riyadh were the subject of this study. Anyone who was eighteen years of age or older could take part in this cross-sectional survey. However, the participants had to fulfill the requirements of being patients who visited any main healthcare facility in Riyadh, Saudi Arabia, if they were 18 years of age or older.

### 2.2 Sampling technique and study sample

A two-stage sampling design was employed to select the study participants. In the first stage, a sampling frame of all 103 primary healthcare centers (PHCCs) in Riyadh, Saudi Arabia, was established. From this frame, 48 PHCCs were selected using a simple random sampling technique to ensure broad geographical coverage across the city. In the second stage, a consecutive sampling approach was utilized to recruit participants from within the selected facilities. Data collectors invited all eligible patients and accompanying family members aged 18 years and older present in the waiting areas during the data collection period to participate. To minimize professional bias, healthcare providers and administrative employees of the facilities were excluded from the study. A total of 14,239 individuals consented to participate and completed the survey. While this approach ensured a random selection of facilities and a large, diverse sample of healthcare attendees, it is important to note that the sampling frame was restricted to users of the primary healthcare system in Riyadh. Furthermore, because the second stage relied on recruiting available attendees in the waiting areas rather than a secondary randomization of patient records, the sample should be considered a representative clinical cohort of PHC users rather than a fully probabilistic community-based sample.

### 2.3 Validation and pilot testing of the research questionnaire

An electronic questionnaire was developed and implemented in Hail, Saudi Arabia, although the survey was not conducted in that region. The research instrument underwent a rigorous multi-step validation process to ensure cultural and linguistic appropriateness. The initial electronic questionnaire was adapted from established English-language literature and subjected to a formal forward-backward translation process by bilingual experts. To establish content and face validity, a focus group meeting was convened with 20 key informants, including clinical experts and practitioners. This group evaluated the instrument for clarity, relevance, and representativeness of the constructs being measured. The Content Validity Index (CVI) for individual items ranged from 0.87 to 1.00, and the overall scale-level CVI (S-CVI) was 0.94, indicating excellent content validity. Following these modifications, a pilot study was conducted among 200 attendees at PHCs to assess the feasibility and comprehensiveness of the questions. The Face Validity Index (FVI) was calculated as 0.91, demonstrating that the items were well-understood and interpreted as intended by the target population. Items identified as unclear, difficult, or non-specific were revised based on feedback from the focus groups to ensure clarity for participants of the main study. The reliability of the instrument was further confirmed through a test-retest procedure with 100 participants, yielding a Pearson correlation coefficient of 0.83, indicating high temporal stability.

### 2.4 Data collection and questionnaire administration

A total of 14,239 participants completed an electronic survey administered via tablets in the presence of trained data collectors to ensure data quality. The questionnaire was structured into three distinct modules. The first section captured sociodemographic characteristics, including age, gender, marital status, educational attainment, and employment status. The second module assessed behavioral factors, such as frequency of physical activity, alcohol consumption, tobacco use, and dietary habits. The third segment focused on clinical history and comorbidities. To ensure clarity in measurement, the primary outcome, T2D, was defined based on a self-reported physician diagnosis. Participants were specifically asked, “Have you ever been told by a doctor or healthcare professional that you have diabetes?” This approach captured individuals with a known clinical diagnosis rather than identifying undiagnosed cases through biochemical measures such as fasting blood glucose or HbA1c. Similarly, other comorbidities, including hypertension, cardiovascular disease, and chronic obstructive pulmonary disease, were recorded based on prior professional medical diagnosis. Obesity was also captured as a self-reported condition rather than through direct anthropometric measurements or calculated Body Mass Index (BMI). Prior to participation, data collectors explained the study objectives and obtained written informed consent. Participation was entirely voluntary, and all data were anonymized to maintain confidentiality.

### 2.5 Statistical methods

Data analysis was performed using SPSS version 29.0. Descriptive statistics, including frequencies and proportions, were calculated for categorical variables. The normality of continuous variables was assessed using histograms and the Kolmogorov-Smirnov test. To identify factors associated with the primary outcome (prevalence of T2D), a systematic modeling approach was employed. For sociodemographic factors, a two-stage approach was used: Univariate analysis followed by a multivariable model where age, education, gender, marital status, employment, and insurance were mutually adjusted. For behavioral risk factors and clinical comorbidities, a three-stage modeling strategy was implemented: Model 1 consisted of univariate analysis assessing the unadjusted relationship of each independent variable with T2D; Model 2 adjusted for the foundational confounders of age and sex; and Model 3 further adjusted for age, sex, and all clinical comorbidities (obesity, hypertension, high cholesterol, and history of heart disease) simultaneously. Results are presented as adjusted odds ratios (AORs) with corresponding 95% confidence intervals (CIs), with p < 0.05 considered statistically significant.

### 2.6 Ethics approval and consent to participate

The study was reviewed by the Ethics Committee of King Fahad Medical City (KFMC) and exempted from requiring ethics approval (approval no. 22-397E). An Approval Letter from the Institutional Review Board (IRB) Chair, however, has been provided.

## 3 Study results

### 3.1 Sociodemographic characteristics of study participants

**Table 1** shows the sociodemographic information of the study participants from Saudi Arabia. The mean age of the population sample is 59.7 years (± 16.6 years). Overall, 43.3% of the research participants were male, and 48.8% were in the 50–75 age range. Of those surveyed, 51.5% reported having completed higher education. Additionally, 51.4% of the study participants reported being employed, and 34.7% were not married. Among the study participants, approximately a quarter (24.3%) had insurance coverage, and 27.7% of them were smokers. **Table 1** shows that 5.2% of the sample was determined to be obese (self-reported), but around two-thirds (60.7%) reported being physically active. The findings showed that 12.4% of the study participants were diabetic and 11% were hypertensive. Furthermore, more than half of the study participants (50.3%) reported driving unsafely on the roads. Approximately one-third of the study participants (35.3%) reported self-screening for blood pressure. However, a smaller proportion screened for blood cholesterol (9.3%) and cardiovascular diseases (3.7%).

**Table 1:** Sociodemographic characteristics of study participants (n=14,239).

Age	Frequency (n)	Percentage (%)
< 50 years	4848	34.0
50 to 75 years	6945	48.8
At least 75 years	2446	17.2
Education		
Primary	572	4.00
Secondary	3937	27.6
Higher Education	7336	51.5
Gender		
Female	8062	56.6
Male	6177	43.4
Marital status		
Single	4939	34.7
Married	9300	65.3
Employment status		
Employed	7317	51.4
Unemployed	6922	48.6
Health status		
Excellent	4798	33.7
Very good	5076	35.6
Good	2815	19.8
Fair	1256	8.80
Poor	294	2.10
Insurance coverage		
Yes	3457	24.3
Smoking		
Yes	3942	27.7
Physical activity		
Yes	8641	60.7
Obesity		
Yes	737	5.2
Diabetes		
Yes	1765	12.4
Hypertension		
Yes	1580	11.1
Driving behavior		
Safe driving behavior	7074	49.7
Unsafe driving behavior	7165	50.3
Screening for blood sugar		
Yes	4397	30.9
Screening for blood cholesterol		
Yes	1324	9.3
Screening for blood pressure		
Yes	5032	35.3
Screening for cardiovascular diseases		
Yes	520	3.7

### 3.2 Socio-demographic factors of diabetes among Saudis at primary healthcare settings in Riyadh

**Table 2** shows the results of the sociodemographic factors associated with Diabetes among Saudis in PHCs settings in Riyadh. Univariate analysis revealed that age, education, gender, marital status, employment status, and insurance coverage were significantly associated with Diabetes. After mutual adjustment in the multivariable model (**Table 2**), a dose-response relationship was found between age and Diabetes. We found that compared to younger participants (< 50 years), those at least 75 years were 6.30 times more likely to be diabetic (AOR: 6.30; 95% CI: 5.11, 7.77). Education was also a strong predictor; compared to individuals who completed primary education, those who completed secondary (AOR: 0.51; 95% CI: 0.41, 0.63) or higher education (AOR: 0.36; 95% CI: 0.29, 0.45) were less likely to have Diabetes by about 49% and 64%, respectively. Also, concerning gender it was found that after adjusting for other sociodemographic factors, males were 1.33 times more likely to be diabetic than females (AOR: 1.33; 95% CI: 1.17, 1.51). In addition, compared to employed Saudi residents, unemployed residents were 1.49 times more likely to be diabetic (AOR: 1.49; 95% CI: 1.30, 1.70). Saudis who had insurance coverage were 1.72 times more likely to be diabetic (AOR: 1.72; 95% CI: 1.51, 1.96) than those without insurance coverage (**Table 2**).

**Table 2:** Demographic and behavioral factors of diabetes among Saudis at primary healthcare settings in Riyadh (n=14,239).

	Univariate analysis				Multivariate analysis		
Predictors		95% CI				95% CI	
Age	OR	LL	UL	P-value	AOR	LL	UL
< 50 years	1			<0.001	1		
50 to 75 years	2.35	2.03	2.72		2.73	2.25	3.30
At least 75 years	7.00	6.01	8.16		6.30	5.11	7.77
Education							
Primary	1			<0.001	1		
Up to High School	0.355	0.29	0.434		0.51	0.41	0.63
College/University	0.251	0.207	0.306		0.36	0.29	0.45
Gender							
Female	1			<0.001	1		
Male	1.24	1.12	1.37		1.33	1.17	1.51
Marital status							
Single	1			<0.001	1		
Married	2.20	1.95	2.48		0.90	0.76	1.06
Employment status							
Employed	1			<0.001	1		
Unemployed	1.49	1.34	1.64		1.49	1.30	1.70
Insurance coverage							
No	1			<0.001	1		
Yes	1.37	1.23	1.53		1.72	1.51	1.96

The multivariable includes age, gender, marital status, education level, employment status, and insurance coverage. All variables in this model are mutually adjusted to determine the independent association of each sociodemographic factor with Type 2 Diabetes; OR, Odds ratio; AOR, Adjusted Odds Ratio; 95% CI, 95% Confidence Interval; LL, Lower limit; UL, Upper Limit.

### 3.3 Behavioral risk factors and comorbidities associated with type 2 diabetes

**Table 3** illustrates the behavioral and clinical factors associated with Diabetes. In the univariate analysis (Model 1), heart disease, hypertension, and high cholesterol were significantly associated with diabetes. However, there was no significant relationship between alcohol use and physical activity with diabetes among Saudis in primary healthcare settings in Riyadh. As per the predefined modeling strategy, only variables meeting inclusion criteria (p < 0.20 or clinical relevance) were retained in Model 2; therefore, alcohol use and physical activity were excluded from the final multivariable model. In Model 2, after adjusting for age and sex, the clinical comorbidities (heart disease, hypertension, and high cholesterol) remained significant predictors of T2D. The final multivariable analysis (Model 3, **Table 3**), which adjusted for age, sex, and all comorbidities simultaneously, revealed that obese participants were 1.39 times more likely to be diabetic (AOR: 1.39; 95% CI: 1.11, 1.75). Furthermore, Model 3 showed that hypertensive patients were 10.42 times more likely to experience diabetes (AOR: 10.42; 95% CI: 9.02, 12.03) and those with high cholesterol were 3.13 times more likely (AOR: 3.13; 95% CI: 2.64, 3.72). Heart disease did not reach statistical significance in the final Model 3 adjustment (AOR: 1.21; 95% CI: 0.94, 1.54).

**Table 3:** Behavioral risk factors and comorbidities associated with type 2 diabetes.

	Model 1 (univariate analysis)				Model 2 (age and sex adjusted)			Model 3: multivariate analysis		
Predictors	OR	95% CI		P-value	AOR	95% CI		AOR	95% CI	
Alcohol use										
No								NA		
Yes	0.67	0.40	1.13	0.134	0.66	0.39	1.12			
Physical activity										
No	1							NA		
Yes	0.95	0.858	1.051	0.32	0.95	0.85	1.06			
Obesity										
No	1.00			<0.001				1		
Yes	4.17	3.55	4.90		4.58	3.85	5.44	1.39	1.11	1.75
Hypertension										
No	1.00			<0.001				1		
Yes	22.44	19.83	25.39		17.82	15.67	20.26	10.42	9.02	12.03
Heart Disease										
No	1			<0.001				1		
Yes	6.41	5.40	7.61		7.38	6.13	8.89	1.21	0.94	1.54
High Cholesterol										
No	1							1		
Yes	11.53	10.20	13.04	<0.001	9.93	8.71	11.3	3.13	2.64	3.70

Model 1, Univariate analysis showing a relationship between one independent variable with Type 2 Diabetes.

Model 2, Adjusted for age and sex.

Model 3, Adjusted for age, sex, and mutually adjusted for co-morbidities including obesity, hypertension, high cholesterol, and history of heart disease.

OR, Odds ratio; AOR, Adjusted odds ratio; 95% CI, 95% confidence interval.

## 4 Discussion

This cross-sectional study was undertaken among residents of the Riyadh region to measure the observed burden of Type 2 Diabetes (T2D) and identify its associated risk factors within this specific population. Overall, the prevalence of T2D was found to be greater than ten percent. While our findings align with the 2024 Saudi General Authority for Statistics (GASTAT) report, which noted a diagnosed diabetes prevalence of approximately 9.1% (12), they are lower than the IDF’s projected 23.1% for the general population. This variation likely stems from our reliance on self-reported physician diagnosis, which captures known cases but may exclude undiagnosed individuals in the community. Concerning the risk factors, it was found that individuals of older age, males, those currently unemployed, and those reporting hypertension, obesity, and high cholesterol had higher odds of T2D. However, highly educated Saudi residents exhibited a lower likelihood of T2D than those with lower educational attainment.

Our study contributed to exploring various epidemiological aspects of T2D among primary healthcare attendees in Riyadh. The findings are consistent with the body of literature and other studies conducted globally. For example, older age, obesity, and education level were a predictive factor for T2D in one study conducted by Aldossari et al., in 2018 (11). While our findings align with global trends, we must exercise caution in generalizing these results to the entire Kingdom, as our sampling was focused on the capital region. The discrepancy between our self-reported prevalence and higher national estimates may be partly explained by the high burden of undiagnosed prediabetes in the Saudi population, which recent systematic reviews estimate to be a major contributor to the overall metabolic burden (5). This underscores the need for biochemical screening to complement self-reported data.

Obesity posing as a risk factor was also supported by previously published literature, stating that obesity could increase the risk of T2D independently of age and other risk factors (13). Likewise, old age appeared to be a universal risk factor for T2D, regardless of geographical location and study setting. For example, studies from Jeddah (14), China (15, 16), and the United States of America (17, 18) suggested that older age is a prevalent risk factor for T2D followed by overweight and obese individuals. The potential mechanisms through which obesity is linked to T2D include poor lifestyle, a lack of physical activity, and the consumption of unhealthy foods. The benefits of these lifestyle interventions are previously established for varying populations such as the Asian (19, 20), Saudi (21), Chinese (22), Americans (23), Swedish, and Finnish populations (24, 25). While these associations are strong, the cross-sectional nature of our data prevents us from concluding that these factors preceded the onset of diabetes (23, 26).

In addition, a strong association was found between high blood pressure and T2D, indicating that diabetic individuals had a higher proportion of hypertensive individuals than those who were non-diabetic. Typically, T2D is thought to contribute to high blood pressure by causing kidney damage, and the co-existence of both diseases synergistically contributes to micro and macrovascular complications (27–29). However, within the cross-sectional framework of this study, the directionality of this relationship remains unclear, and we cannot definitively state whether hypertension preceded T2D or vice versa. It is equally plausible that the shared lifestyle factors leading to hypertension also contribute to T2D, or that the management of one condition increased the clinical detection of the other. One plausible explanation for the observed relationship could be the use of certain antihypertensive medications, such as thiazide diuretics or non-selective beta-blockers, which have been shown to alter glucose metabolism and potentially increase the risk of T2D (30). Given these complexities, the association observed in our cohort underscores the high burden of comorbidity but must not be interpreted as a causal link.

However, higher education was found to be a protective factor against T2D. These findings are consistent with previous studies across the world. Referencing a study conducted in China, it was revealed that men with lower education were at higher risk of T2D than men with a higher education level (31). Similarly, findings from Europe (32), Ethiopia (33), Sweden (34), and Bangladesh (35) also found consistent results where researchers concluded that higher education was a preventative factor for developing T2D. These findings collectively suggest education may increase the level of awareness and knowledge of an individual on how one can adopt a healthy lifestyle to be protected from T2D.

### 4.1 Strengths and limitations

This study possesses several key strengths. Primary among these is the implementation of a two-stage sampling design, which involved the random selection of 48 primary healthcare centers followed by the consecutive sampling of a large participant pool. The remarkably large sample size enhances the precision of our estimates and allows for a robust multivariable logistic regression analysis to control for various confounders. Additionally, the study utilized a questionnaire with high test-retest reliability and established content validity indices, reinforcing the internal consistency of the data collection. However, certain limitations must be acknowledged. A primary limitation is that the sampling frame was restricted to attendees of PHCCs within the Riyadh region. Because this was a facility-based sample rather than a random community-based household survey, it is subject to selection bias; individuals who attend healthcare facilities may have different health-seeking behaviors or a higher prevalence of comorbidities compared to the general public. Consequently, throughout this manuscript, we have revised our terminology to specify that these findings represent PHC attendees in Riyadh and may not be directly generalizable to the wider Saudi Arabian population. Second, the reliance on self-reported comorbidities and health status, rather than biochemical confirmation (such as HbA1c or fasting glucose) or objective anthropometric measurements, introduces the potential for measurement and recall bias. This is particularly evident in the reported obesity prevalence of 5.2%, which is likely an underestimation due to self-reporting bias. The observed obesity prevalence of 5.2% is significantly lower than reported national averages (often exceeding 30%). This discrepancy is primarily due to the study’s reliance on self-reported obesity status. Participants were asked to self-identify their status rather than having their Body Mass Index (BMI) calculated through objective anthropometric measurements of height and weight. This methodology likely introduced significant self-report bias, as individuals may under-recognize or under-report their weight status, leading to an underestimation of the association between obesity and T2D. Due to the cross-sectional nature of the study design, we cannot establish temporal relationships or rule out reverse causation, particularly concerning the association between hypertension and T2D. For instance, individuals already diagnosed with T2D may have modified their dietary habits or physical activity levels prior to the survey, which could mask the true strength of the association between these behavioral factors and the disease. Consequently, our results should be interpreted as a snapshot of associations rather than evidence of causal pathways. Future research employing prospective cohort designs is necessary to determine definitive temporal links between these risk factors and the onset of T2D.

### 4.2 Conclusion

Type 2 Diabetes (T2D) is a serious public health concern showing a high prevalence among primary healthcare attendees in Riyadh. Given the constellation of risk factors associated with T2D, urgent public health actions are required to intervene at the population level. The government of Saudi Arabia needs to prioritize its residents who are less educated, male, unemployed, older-aged, obese, and hypertensive. By focusing on modifiable risk factors through evidence-based lifestyle interventions and early screening, the clinical burden of T2D can be mitigated.

## Data Availability

The original contributions presented in the study are included in the article/supplementary material. Further inquiries can be directed to the corresponding author.
